# Effect of ocean outfall discharge volume and dissolved inorganic nitrogen load on urban eutrophication outcomes in the Southern California Bight

**DOI:** 10.1038/s41598-023-48588-2

**Published:** 2023-12-13

**Authors:** Minna Ho, Fayçal Kessouri, Christina A. Frieder, Martha Sutula, Daniele Bianchi, James C. McWilliams

**Affiliations:** 1 Southern California Coastal Water Research Project Authority, Costa Mesa, CA 92626 USA; 2 Department of Atmospheric and Oceanic Sciences, University of California, Los Angeles, CA 90095 USA

**Keywords:** Marine chemistry, Carbon cycle, Element cycles, Fluid dynamics

## Abstract

Climate change is increasing drought severity worldwide. Ocean discharges of municipal wastewater are a target for potable water recycling. Potable water recycling would reduce wastewater volume; however, the effect on mass nitrogen loading is dependent on treatment. In cases where nitrogen mass loading is not altered or altered minimally, this practice has the potential to influence spatial patterns in coastal eutrophication. We apply a physical-biogeochemical numerical ocean model to understand the influence of nitrogen management and potable wastewater recycling on net primary productivity (NPP), pH, and oxygen. We model several theoretical management scenarios by combining dissolved inorganic nitrogen (DIN) reductions from 50 to 85% and recycling from 0 to 90%, applied to 19 generalized wastewater outfalls in the Southern California Bight. Under no recycling, NPP, acidification, and oxygen loss decline with DIN reductions, which simulated habitat volume expansion for pelagic calcifiers and aerobic taxa. Recycling scenarios under intermediate DIN reduction show patchier areas of pH and oxygen loss with steeper vertical declines relative to a “no recycling” scenario. These patches are diminished under 85% DIN reduction across all recycling levels, suggesting nitrogen management lowers eutrophication risk even with concentrated discharges. These findings represent a novel application of ocean numerical models to investigate the regional effects of idealized outfall management on eutrophication. Additional work is needed to investigate more realistic outfall-specific water recycling and nutrient management scenarios and to contextualize the benefit of these management actions, given accelerating acidification and hypoxia from climate change.

## Introduction

Worldwide, climate change is increasing the frequency and severity of drought^1^. In drought-prone areas, one strategy to secure adequate water resources is to shift towards increased water conservation and recycling, including the beneficial reuse of municipal wastewater. Ocean wastewater discharges are an attractive target for water recycling and reuse. In California, an estimated 417 billion gallons of wastewater effluent are discharged annually to the Pacific Coast and San Francisco Bay^2^. Water recycling for non-potable uses is already widespread and involves diverting of effluent from the outfall for industrial uses or irrigation of public spaces. This results in a net decrease in effluent volume and constituent loads to coastal zones^3^. However, potable water recycling in this region, which involves the use of treatment technologies such as microfiltration and reverse osmosis to remove contaminants to potable standards, can result in the return of the reverse osmosis concentrate to the ocean outfall, such that constituent mass loading is conserved but with lower volumes and higher concentrations. Methods to treat this reverse osmosis concentrate exist, but are expensive. Sending the concentrate to the outfall is a cost-effective means of disposal^4, 5^. While potable water recycling alone does not necessarily imply significant changes in nitrogen loading, other technologies and practices exist that can change DIN species (e.g., from ammonium to nitrate) and/or reduce nitrogen to meet operational needs and treatment standards for individual dischargers. The environmental effects of potable water recycling on ocean discharges are unclear, especially if the reduction in volume has the potential to change spatial patterns in the dispersion of associated pollutants^6, 7^.

Coastal eutrophication, the accelerated accumulation of organic matter^8^, is a global environmental problem that has the potential to be influenced by potable water recycling. Eutrophication is the outcome of anthropogenically enhanced primary productivity, resulting in coastal acidification and hypoxia^9, 10^. Marine environments are typically nitrogen limited, so nitrogen pollution is a major cause^11^. Globally, municipal wastewater is one of the major sources of coastal nitrogen pollution^12^, and the level of wastewater treatment controls the concentration and loads of dissolved inorganic nitrogen (DIN; ammonium, NH$_{4}^{+}$; nitrate, NO$_{3}^{-}$; and nitrite, NO$_{2}^{-}$) that are discharged to the ocean. In California, many coastal wastewater utilities discharge effluent that has received primary or secondary treatment, with values typically in the range of 30-40 mg/L DIN, most of which is in the form of ammonium^13^. Phytoplankton readily uptake and assimilate ammonium compared to nitrate, and thus may lead to exacerbated eutrophication^14^. With advanced nitrogen removal methodologies, DIN can drop to ranges of 3-5 mg/L, most of which is typically in the form of nitrate^15^. Plant upgrades that would be required to achieve these levels would come at high capital and operational costs, and other potential negative environmental impacts^16^.

Changes to outfall effluent characteristics from potable water recycling have the potential to alter the spatial and temporal patterns of dispersion of wastewater^6, 7^, key factors that control the rate of eutrophication in coastal waters^10^. Marine outfalls discharge wastewater that is designed to encourage rapid effluent mixing sufficient to maintain a submerged waste field below the photic zone, allowing for strong dilution and dispersal. Altering outfall discharge volume and density has the potential to influence the plume rise height, spreading and dilution^17, 18^, which has the potential to impact where and when algal productivity is enhanced and the extent to which eutrophication outcomes develop^10^. Most plume modeling has been nearfield and focused on end of pipe requirements^19–22^. High resolution coupled physical-biogeochemical models have not been routinely used for ocean outfall environmental effect studies^10, 23^, so their use can not only inform our understanding of regional-scale environmental effects but also where additional focus on outfall management might be beneficial.

**Figure 1:**
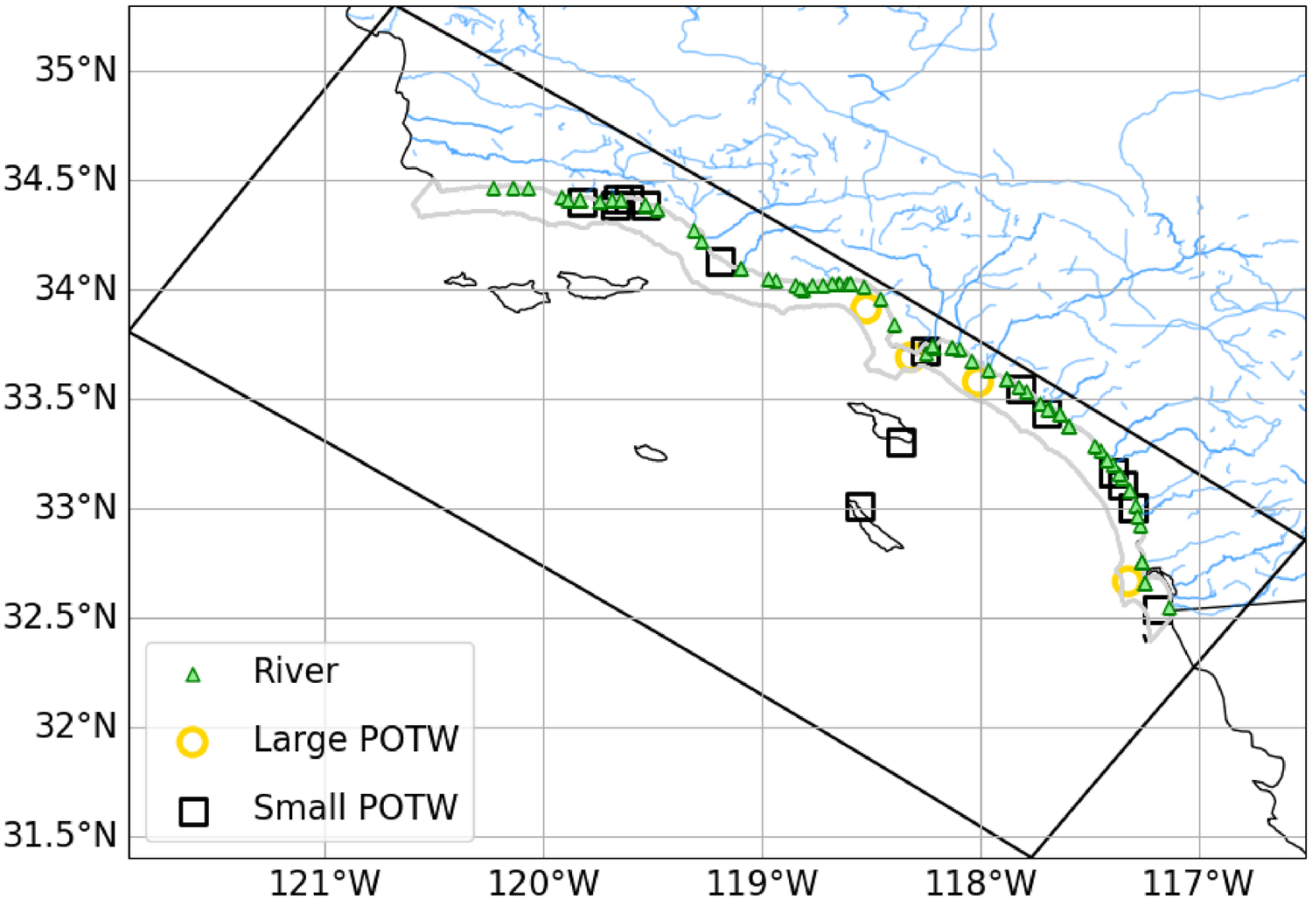
Map of the Southern California Bight model domain (black box, also known as L2 domain, dx = 300 m) with location of model rivers and POTWs terrestrial sources. Gray contour indicates the 15 km coastal band. See Kessouri et al. (2021)^10^, Figure 3, for a zoomed version of the location of the coastal sources. Note that use of “Bightwide” throughout this study refers to the entire model domain, shown here. Generated with Python 3.8.17 (https://www.python.org/downloads/release/python-3817/) using library Cartopy 0.21.0 (https://pypi.org/project/Cartopy/0.21.0/).

The Southern California Bight (SCB), an upwelling dominated open embayment located on the U.S. Pacific West Coast, represents an excellent location for a case study on the effects of potable water recycling on coastal eutrophication for several reasons. First, the SCB has a large coastal population of 22 million people that create a combination of point and non-source discharges to this marine ecosystem^13^. Point sources discharged to ocean outfalls dominate coastal nitrogen exports in this region, representing 70% of freshwater volume and 92% of nitrogen loads, the majority of which are discharged from 23 publicly owned treatment works (POTWs) to 19 ocean outfalls (Fig. 1). Second, documentation exists that these coastal nitrogen exports are causing eutrophication, including increase in coastal algal productivity of up to 150% accompanied by subsurface oxygen (O$_2$) and dissolved inorganic carbon (DIC) losses^10, 24^. In turn, these chemical changes are causing seasonal compression of habitat for pelagic calcifying zooplankton and aerobic taxa^25^. Third, strong interest exists in potable water recycling at the statewide and local level; California has released a new plan to dramatically increase water reuse goals of 260 billion gallons of potable water by 2030, with municipal wastewater representing the largest resource available for water recycling^26^. Among the 23 POTWs that discharge to ocean outfalls in the SCB, four POTWs discharge greater than 50 million gallons per day (hereto referred to as large POTWs). Collectively they represent 86% of the volume discharged by ocean outfalls to the SCB^13^. One of these large utilities (Orange County Sanitation District; OC San) has already implemented a joint project with OC Water District to recycle wastewater to indirect potable water reuse^27^, while the other three large utilities have initiated planning. Fourth, the SCB has substantial observational and numerical modeling research assets, which have been effectively channeled towards the development and validation of a coastal numerical model capable of disentangling the relative effects of local anthropogenic versus global climate change effects on eutrophication and associated outcomes^28–31^. The numerical model has been utilized to document the effects of land-based source of nutrients on marine algal productivity, acidification, and oxygen loss^10, 24^, and to translate the changes in subsurface pH and oxygen to predictions of changes to habitat capacity for epipelagic marine calcifier and aerobic taxa^25^.

In this study, we utilize this coastal numerical model to examine a suite of six idealized scenarios to test the effects of potable wastewater recycling, in combination with effluent DIN management, on coastal eutrophication outcomes. Specifically, we seek to answer: (1) What are changes in eutrophication state (O$_2$, pH) and rate (respiration, NPP) variables resulting from these experimental alterations? (2) What are the implications of these experimental alterations in eutrophication outcomes on associated biological effects? And (3) how does altered effluent characteristics resulting from progressive amounts of water recycling and/or DIN management change transport of DIN?

## Results

### Effects of nitrogen management alone

**Figure 2:**
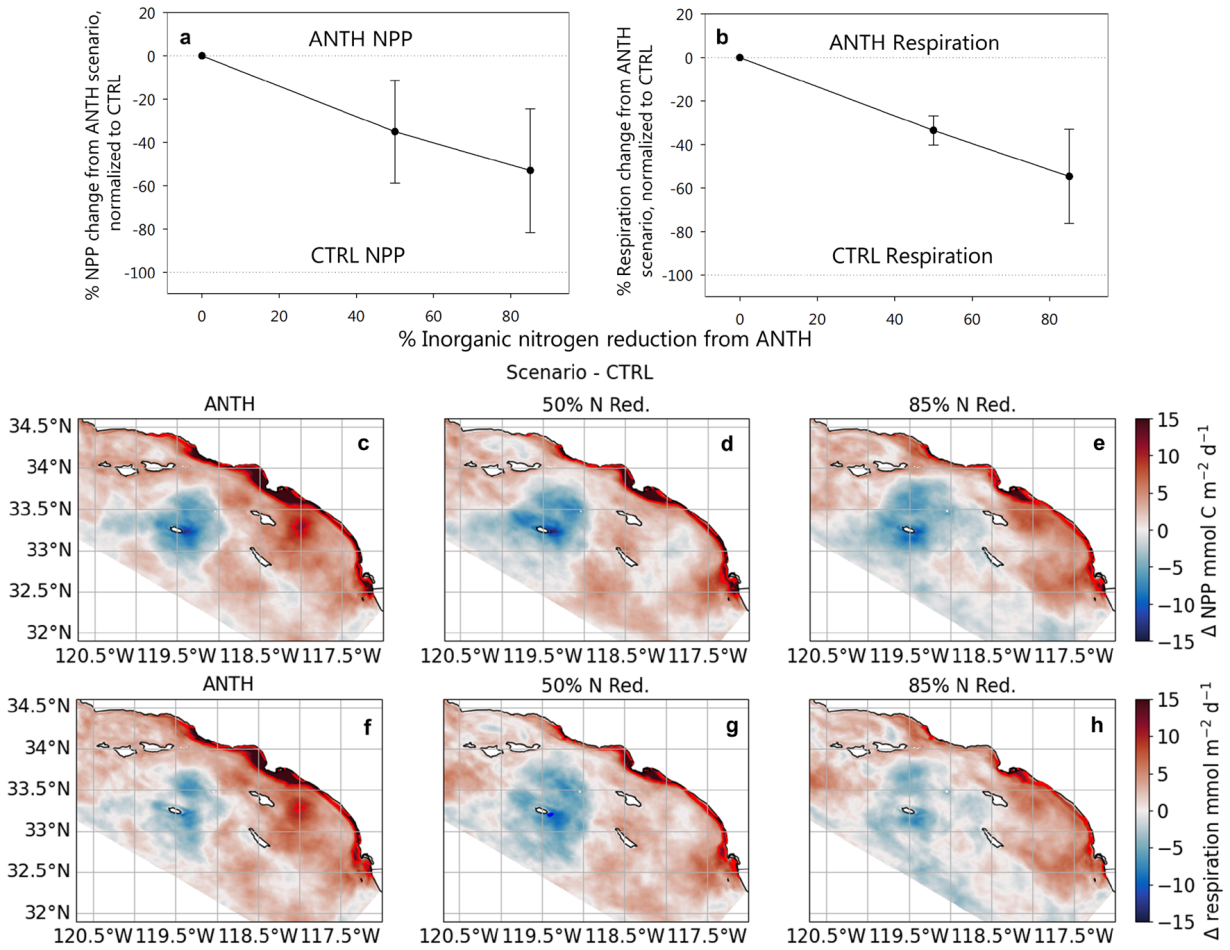
Change in (**a**, **c**-**e**) integrated NPP over the top 100 m and (**b**, **f**-**h**) average respiration over the top 200 m from the CTRL for scenarios ANTH, 50% and 85% DIN reduction, shown as (**a**, **b**) Bightwide averages and (**c**-**h**) average maps over 2 years (November 2015–October 2017). For (**a**) and (**b**), data points represent mean Bightwide % change, normalized over the % difference between ANTH - CTRL, with the vertical line as standard error of mean (*n* = 4 years; 1998, 1999, 2016, 2017). Panels (**c**-**e**) and (**f**-**h**) show changes in NPP and respiration, respectively, from the CTRL scenario for ANTH, 50%, and 85% DIN reduction for 2016-2017. Red colors indicate increase in NPP; blue colors decrease in NPP relative to CTRL. Red contours indicate +10 mmol m$^{-2}$ d$^{-1}$. See Supplementary Fig. S1 for 1998-1999 average maps.

Reduction of DIN loading alone, without additional potable water recycling, shows a strong correlation with NPP and respiration, reversing both the overall magnitude of change (Fig. 2a-b) across the Bight as well as its spatial footprint (Fig. 2c-h). Relative to the 2015-2017 loads in the ANTH scenario, average integrated NPP over the top 100 m declines a mean of 35% (*n* = 4 years, range +34 to -88%) at 50% DIN reduction to a mean of 53% (range +0.6 to -141%) at 85% reduction (Fig. 2a). This declining trend in NPP is strongly associated with changes in Bightwide respiration rates, both in mean Bightwide trends (Fig. 2b) as well as spatial coherence of changing rates (Fig. 2c-h; Supplementary Fig. S1).

Lower DIN loads result in less available N for phytoplankton uptake and consequently smaller magnitudes of NPP and respiration rates, as well as decreased spatial distribution of elevated rates compared to ANTH (Fig. 2; Supplementary Fig. S1). Accordingly, the biological responses between the 50% and 85% reduction cases are distinct. Across the entire Bight, 50% and 85% DIN reduction reduce the average area of elevated NPP (respiration rate) by 11% (9%) and 25% (20%), respectively. The decrease of biological response with DIN reduction is most notable along the coast (Fig. 2c-h; Supplementary Fig. S1). Inshore, defined as the first 15 km from the coastline, the average NPP (respiration rate, averaged over 200 m) decreases by 2.9 (2.9) and 4.1 (3.4) mmol m$^{-2}$ d$^{-1}$ at 50% DIN reduction and 85% DIN reduction, respectively. These 2016-2017 time-averaged inshore NPP (respiration) declines reach up to 9.6 (7.6) at 50% DIN reduction and 17.3 (12.8) mmol m$^{-2}$ d$^{-1}$ at 85% DIN reduction (95th percentile value).

**Figure 3:**
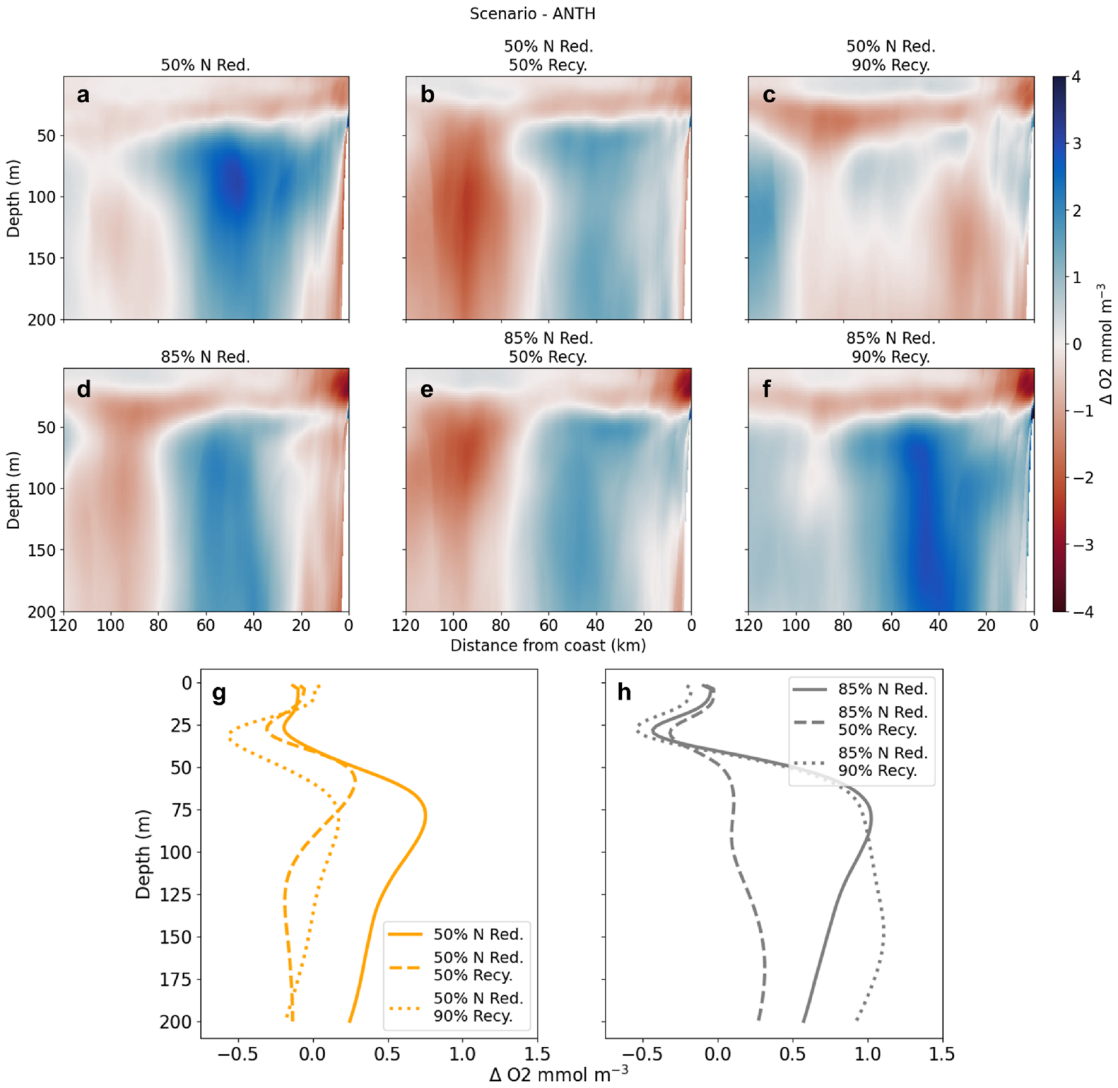
(**a**-**f**) Bightwide alongshore-averaged vertical cross sections (0-200 m) in O$_2$ change relative to ANTH over the 2 years as a function of distance from the coast (x-axis). Red shade denotes decreased O$_2$, while blue shade denotes increase. (**g**-**h**) Bightwide average vertical profiles (0-200 m) of net change in O$_2$ relative to ANTH, where positive is an increase and negative is a decline in O$_2$.

Declining NPP and respiration translate to increases in subsurface oxygen and pH, which are visible in Bightwide absolute changes (Supplementary Table S1) as well as vertical cross sections and profiles (Fig. 3a,d,g-h; Supplementary Fig. S2). Note a decrease in oxygen occurs near the shore and surface in the 85% N reduction cases (Fig. 3d-f) because NPP is decreased. Decreased nutrient load from POTWs leads to reduced NPP, which decreases the oxygen production at the surface, leading to the negative oxygen gradient for these scenarios compared to ANTH.

**Figure 4:**
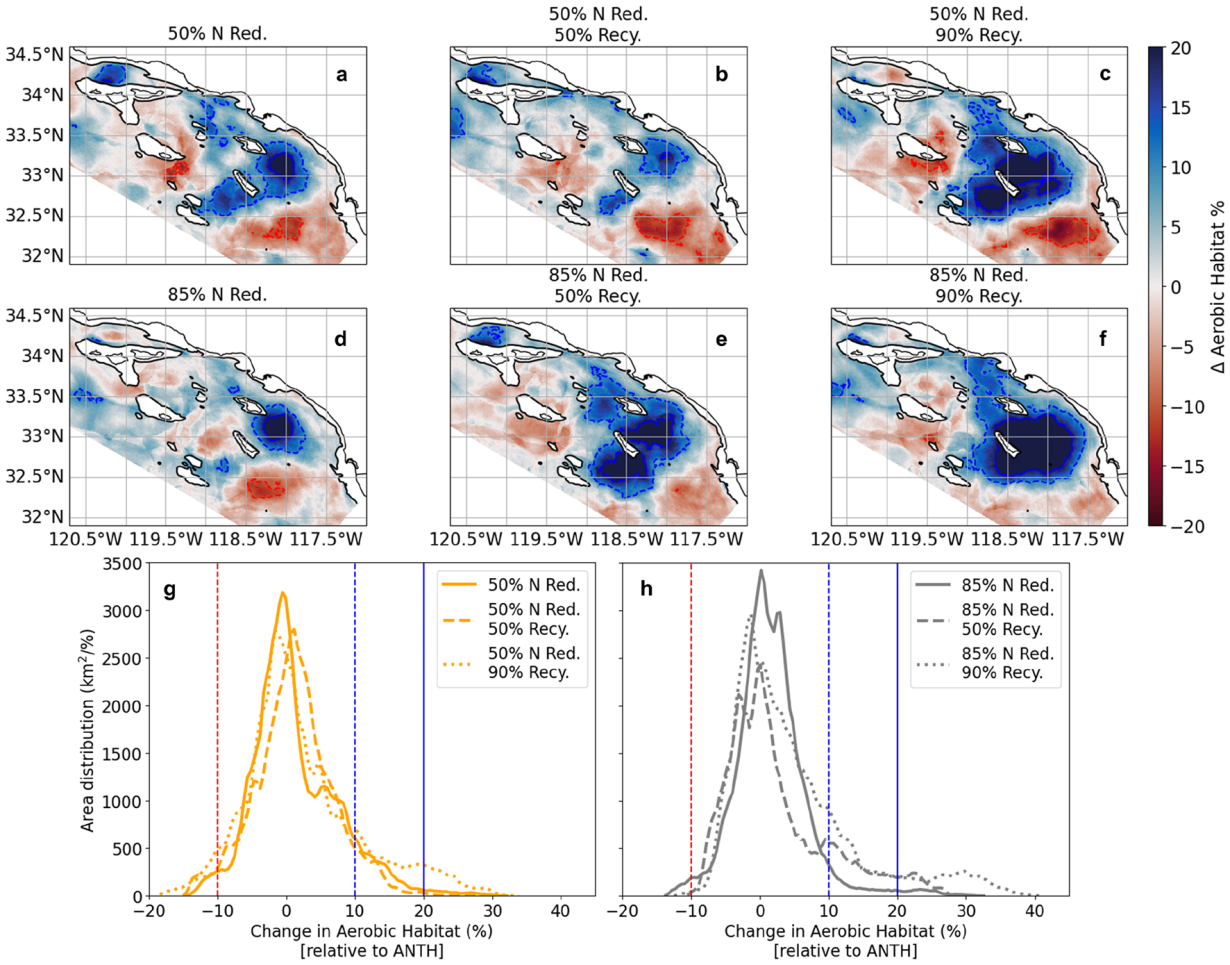
(**a**-**f**) Averaged August-October (n = 6 months; 2016-2017) % change in aerobic habitat, relative to ANTH, for all six DIN management and water recycling scenarios. Red colors and contours indicate habitat contraction; blue colors and contours habitat expansion (dashed line: ±10%; solid line: ±20%). Black contours show the 200 m isobath; depths shallower than 200 m are masked out to highlight pelagic environments. (**g**-**h**) Distributions of the total areal extent as a function of change in aerobic habitat capacity (km$^2$/%) for each scenario relative to ANTH. Vertical red and blue lines map to contour values in (**a**-**f**).

For most of the year and for most of the Bight ($\sim$80%), there are negligible changes in the vertical thickness of aerobic and calcifier habitat from nutrient management. However, from August to October^25^, the increases in subsurface O$_2$ concentrations and pH translate to an expansion in the vertical thickness of aerobic and marine calcifier habitat (Fig. 4, Supplementary Fig. S3). The changes in both aerobic and calcifier habitat thickness are most pronounced in a region southeast of Catalina Island and within the Santa Barbara Channel.

Interannual variability in these responses are evident from the ranges of change in NPP. The years 1999 and 2017 are high NPP years (ANTH = 111.1 and 90.2 mmol C m$^{-2}$ d$^{-1}$, respectively), which coincide with lower reductions in NPP (as little as -7% at 85% DIN reduction). In contrast, the years 1998 and 2016 have relatively lower NPP (ANTH = 74.2 and 86.5 mmol C m$^{-2}$ d$^{-1}$, respectively) and higher reductions in NPP (as much as -115% at 85% DIN Reduction) (Fig. 2a-b).

### Effects of nitrogen management with water recycling

**Figure 5:**
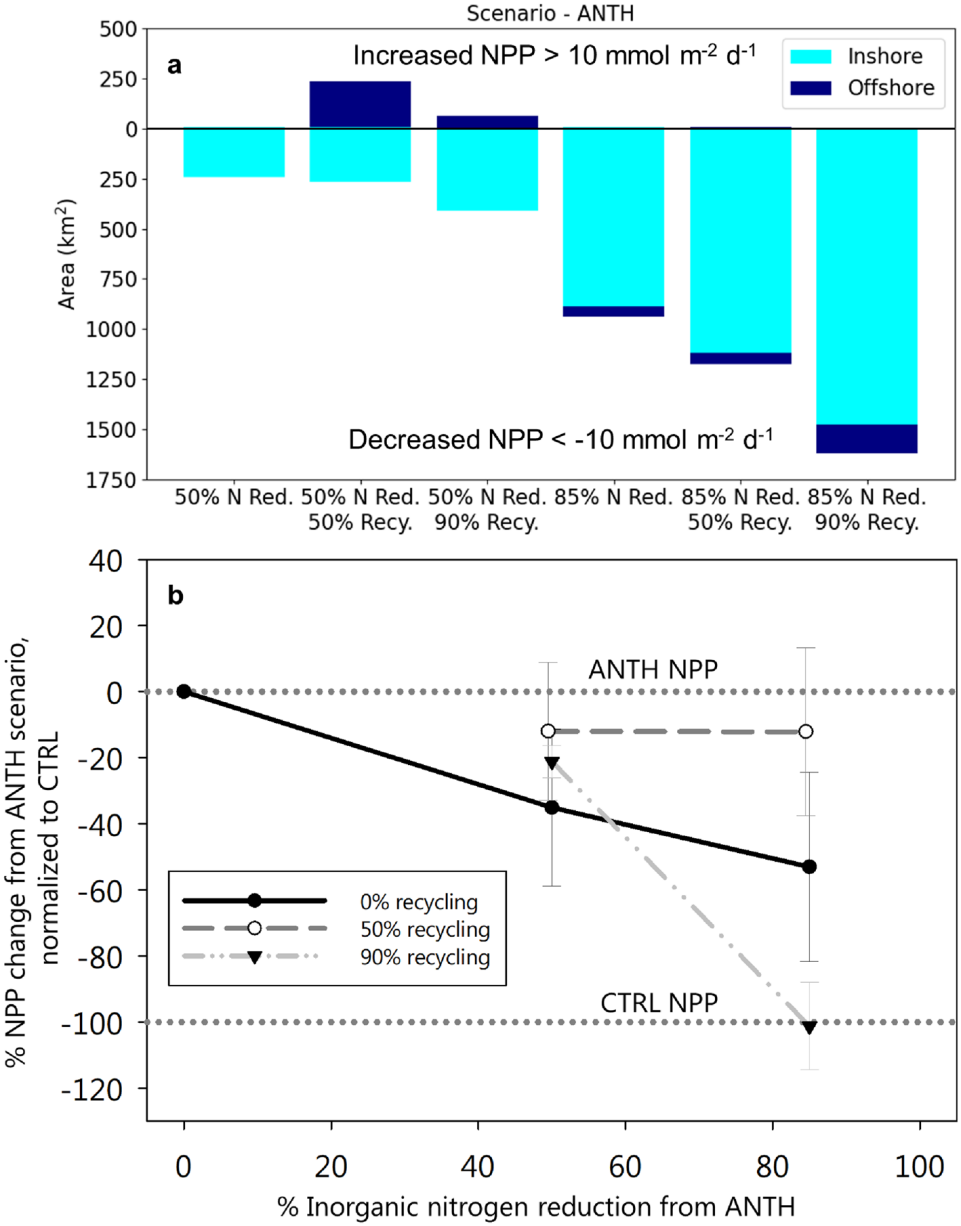
(**a**) Area (km$^{2}$) within model domain exhibiting considerable values in NPP ($> +10$ or $< -10$ mmol m$^{-2}$ d$^{-1}$) across the six scenarios inshore (coastal band of 15 km; see Fig. 1) and offshore (beyond coastal band), relative to ANTH, illustrating that at 50% nitrogen reduction, considerable values in NPP are more common offshore, while at 85% reduction, considerable values in offshore productivity were extinguished. (**b**) % change in Bightwide mean and range in NPP (*n* = 2 years; 2016-2017) with vertical line as standard error of mean.

**Figure 6:**
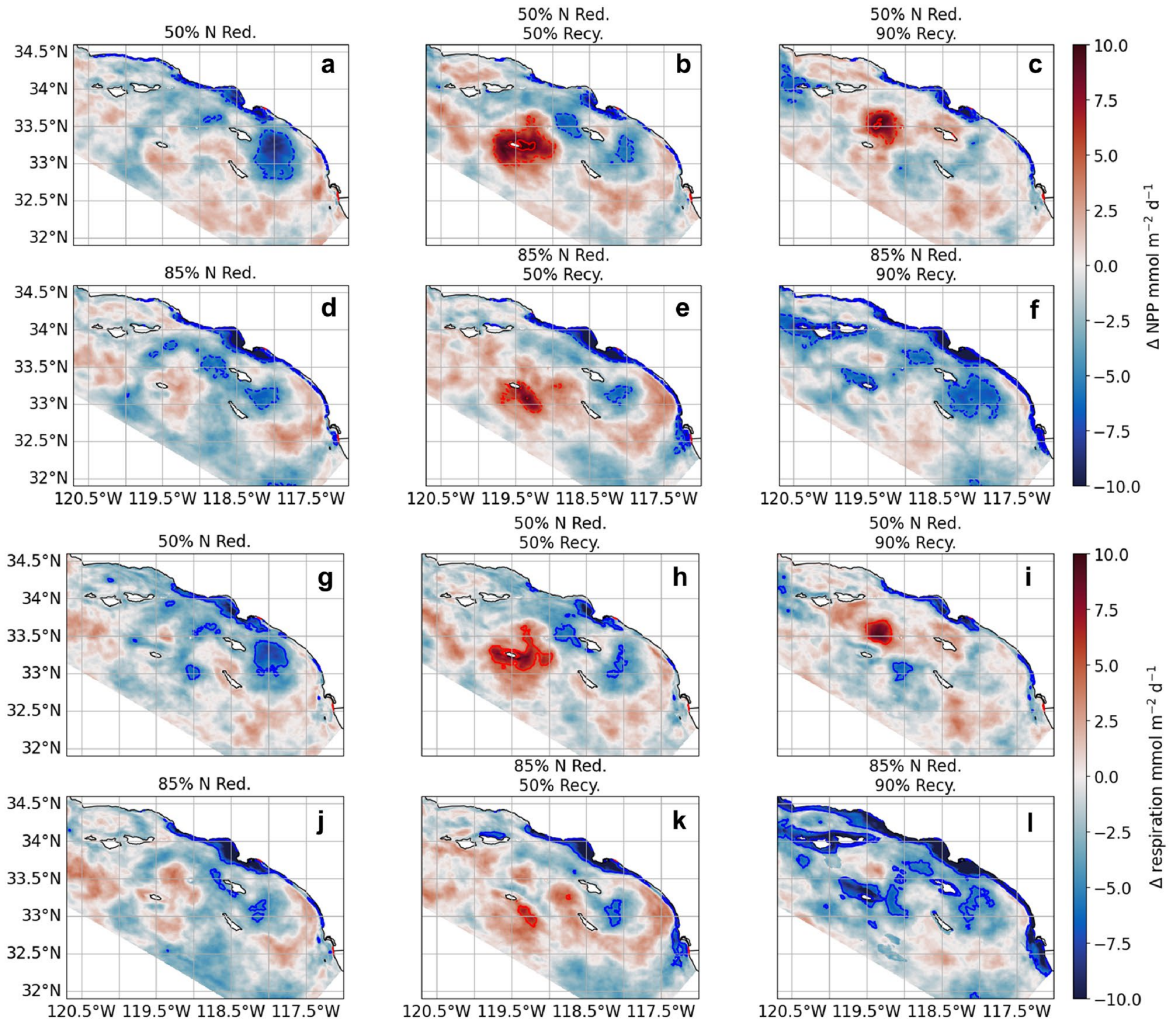
Average 2-year (2016-2017) mean change in (**a**-**f**) NPP and (**g**-**l**) respiration rate relative to ANTH for all six DIN management and water recycling scenarios. Red colors indicate an increase in NPP and respiration, blue colors a decrease. Red dashed and solid contours show respectively 5 and 10 mmol m$^{-2}$ d$^{-1}$ increases in NPP and respiration. Blue dashed and solid contours show respectively 5 and 10 mmol m$^{-2}$ d$^{-1}$ decreases in NPP and respiration.

On average, across scenarios of moderate to extreme water recycling, the mean NPP within the 50% DIN reduction is not markedly different, indicating that water recycling does not appreciably alter carbon cycling Bightwide (Fig. 5b). However, the treatment of 85% DIN reduction, 90% recycling has much higher declines than 0% and 50% recycling treatments compared to ANTH (Fig. 5b, Supplementary Table S1). This scenario marks the greatest reduction in inshore mean and maximum NPP and area of average elevated NPP with a reduction of 6.7, 23 mmol m$^{-2}$ d$^{-1}$ and 37%, respectively, with its 85% DIN reduction, 0% recycling counterpart at 4.1, 17 mmol m$^{-2}$ d$^{-1}$ and 25% reduction, respectively. In addition, even as nitrogen management reduces the overall footprint of NPP and respiration (Fig. 5a; Fig. 6), at intermediate levels of DIN removal, water recycling appears to shift and intensify NPP (Fig. 6b,c,e,h,i,k) causing an increased area of considerable values in positive NPP (>+10 mmol m$^{-2}$ d$^{-1}$) (Fig. 5a). Decreased NPP and respiration rates (<-10 mmol m$^{-2}$ d$^{-1}$) are consistently close to shore in DIN reduction scenarios. Considerable values in increased NPP and respiration are more common offshore than inshore (Fig. 6b,c,h,i). At 85% DIN reduction, any positive considerable values in offshore productivity are extinguished and considerable values in NPP declines are notably enhanced offshore (Fig. 5a; Fig. 6e,f,k,l).

These changes in NPP and respiration translate to a gradient of changes in subsurface O$_2$ and DIC loss (Supplementary Table S1, Fig. 3; Supplementary Fig. S2) across the Bight. Increases to subsurface pH and O$_2$ regimes are most notable at ranges of 30-80 km offshore under progressive DIN removal (Fig. 3a,d; Supplementary Fig. S2). At 50% recycling, regardless of DIN management, oxygen consistently increases in the 0-80 km range and areas further offshore experience declines (Fig. 3b,e; Supplementary Fig. S2). For 50% DIN reduction without additional recycling, the reversal of subsurface pH and O$_2$ loss occurs notably in the 50-125 m depth range (Fig. 3g-h; Supplementary Fig. S2), while at 50% recycling, O$_2$ and pH show gains in the 40-75 m depth range, then declines after 75 m. For 85% N reduction at all levels of reclamation, depths below 40 m show an increase in O$_2$ and pH.

The benefits of nutrient management for expanding aerobic and calcifier habitat capacity are maintained under all scenarios of wastewater recycling, and the benefits continue to occur in the same regions and at the same time of year (August-October) as seen in the nutrient reduction with no potable water recycling scenarios (Fig. 4; Supplementary Fig. S3). With progressive DIN removal, it appears that the benefits of increasing water recycling are even more apparent. Both (1) the spatial area undergoing habitat expansion and (2) the magnitude of that expansion is greatest for both habitat capacity metrics in the scenarios with the most water recycling (Fig. 4; Supplementary Fig. S3). As an example, the areal extent of aerobic habitat undergoing more than 20% habitat expansion (relative to ANTH) is approximately 500 km$^2$ for scenarios with 50% and 85% DIN reduction without water recycling as well as 50% water recycling at 50% DIN reduction. The areal extent increases to about 3,000 km$^2$ for 50% DIN reduction with 90% recycling and 85% DIN reduction with 50% recycling. At 85% DIN reduction and 90% water recycling the areal extent of greatest habitat expansion is the largest at more than 5,500 km$^2$. While the patterns and trends in habitat change are consistent for both habitat metrics, the benefits of nutrient management for aerobic habitat thickness are greater than those predicted for calcifier habitat thickness.

### Mechanisms controlling simulated patterns of biogeochemical change

**Figure 7:**
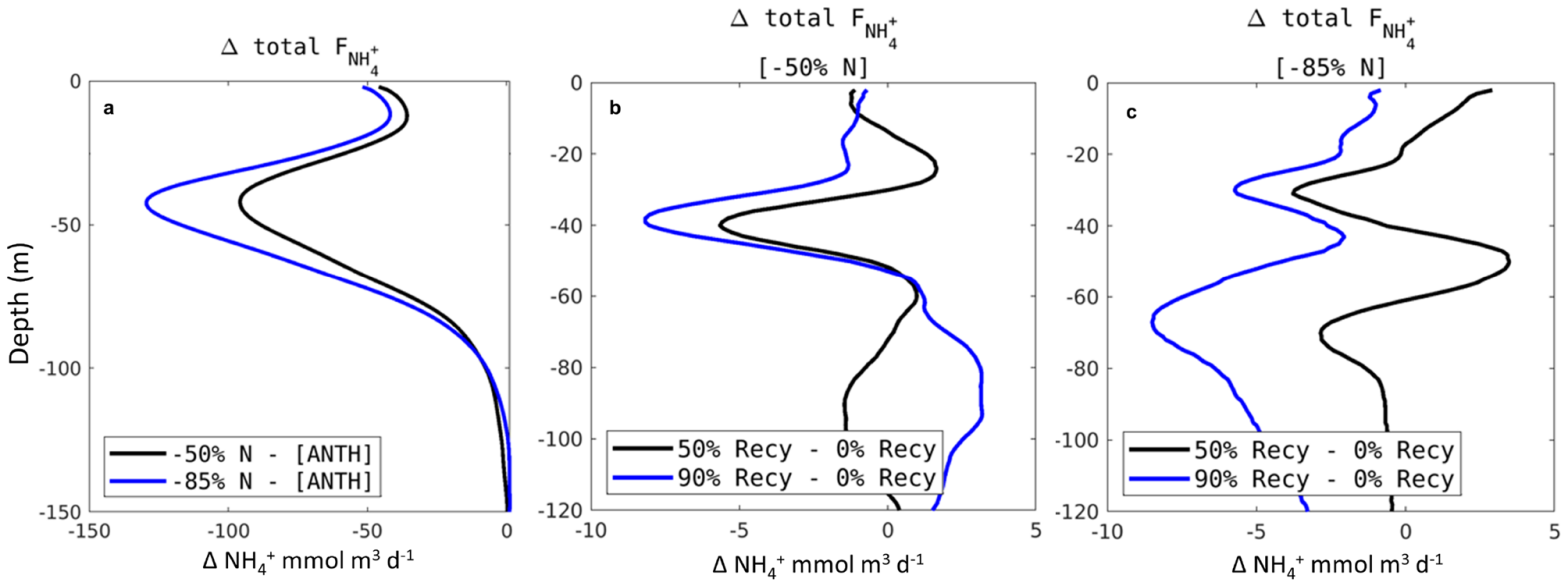
Alongshore (along 200 m isobath) 2-year average profiles of the change in offshore transport of ammonium for (**a**) Scenarios 1, 4 - ANTH, where negative values indicate decrease in offshore export compared to ANTH, (**b**) Scenarios 2, 3 - Scenario 1, and **c** Scenarios 5, 6 - Scenario 4. (**b**, **c**) Positive values indicate more offshore export compared to the respective nutrient management scenario; negative values indicate less.

Altered nitrogen loading and changes to effluent volume and density under progressive water recycling modify spatial patterns and horizontal transport of DIN. A change assessment of the horizontal ammonium flux shows that offshore export (across the 200 m isobath) decreases linearly with the reduction of nitrogen inputs compared to ANTH (Fig. 7). At 50% DIN reduction, the total export flux is reduced by 25 mmol m$^{-3}$ d$^{-1}$. At 85% DIN reduction, the total export flux is reduced by 29 mmol m$^{-3}$ d$^{-1}$.

The change assessment of the ammonium export from volume reduction shows a minor difference (Fig. 7). The largest change is observed in the shape of the export. At 50% N reduction, the export at 50 and 90% recycling decreases at 40 m and increases deeper starting from 60 m depth. The exception to this is the 85% DIN reduction, 90% recycling scenario, where the profile shows a significant overall reduction of the export in the water column.

## Discussion

Management of coastal nutrient exports can be viewed as a potential local adaptation strategy to increase coastal ecosystem resilience to climate change^32, 33^, in essence to “buy time.” At the same time, wastewater and stormwater discharges to the ocean are increasingly eyed as a resource to support water resource security. Only numerical modeling can help to tease apart the potential environmental benefits and risks of combining these strategies. Our findings represent a novel application of ocean numerical models to investigate an idealized case study of the combined regional effects of nitrogen management and water recovery on coastal eutrophication. In this study, we found that NPP, respiration rate, and pH and oxygen loss decline proportionally with outfall DIN load reductions. This translates to gains in habitat volume for pelagic calcifying zooplankton and aerobic fish habitat. In contrast, the lower ecosystem exhibited a non-linear response to progressive amounts of wastewater recycling. We document that decreasing ocean effluent volume under constant nitrogen loading (i.e., increased DIN concentration), without any outfall specific modifications, strongly influenced the spatial and temporal patterns of dispersion of wastewater and its influence on eutrophication processes^6, 7, 10^. In intermediate nitrogen reduction scenarios, advanced water recycling causes spatially localized elevated values in NPP, oxygen loss, and acidification within a smaller footprint achieved by nitrogen management alone. These elevations are diminished in the 85% DIN reduction scenarios across all levels of wastewater recycling, indicating that nitrogen management could mitigate eutrophication risk even under more concentrated ocean discharges, albeit at substantial costs. Thus, our findings underline the importance of further investigating how alteration of freshwater flows can alter the fate, transport, and environmental effects of ocean outfall contaminant plumes in coastal marine waters^34, 35^, given that global wastewater inputs of N are predicted to increase in the future^36^, and these wastewater inputs will increasingly be targeted for water recovery.

Our findings that DIN reductions from 50 to 85% produce a decline in NPP, an increase in subsurface oxygen and pH, and an expansion of habitat for marine calcifier and aerobic taxa, relative to the current day scenario (ANTH), are credible for two reasons. First, there is strong evidence that coastal ecosystems are nitrogen-limited in this^28^ and other regions. Three decades of global efforts to reduce anthropogenic N inputs worldwide have been successful to move eutrophic coastal ecosystems toward recovery, as recent evidence for Danish nearshore waters^37^, Tampa Bay^38^, Chesapeake Bay^39, 40^, the Wadden Sea^41, 42^, and areas within the Baltic Sea^43^, among others, demonstrate^44^. Second, ROMS-BEC model skill assessment has demonstrated high fidelity in multi-year comparisons of simulations with observations of NPP, Chlorophyll-a, and O$_2$ over multiple years under variable terrestrial nutrient loading, lending credibility to an application for this purpose^30^. Model skill relative to observations continues to be assessed for ROMS-BEC simulations of ANTH, including for the simulation period utilized in this study.

Our findings suggest that DIN reductions could improve habitat capacity for marine calcifier and aerobic taxa, thus increasing resiliency in the face of declining trends. Others have found that habitat compression can occur from anthropogenic CO$_2$ emissions^45, 46^ or from local anthropogenic nutrient inputs^47–49^. Frieder et al. (submitted)^25^ analyzed the difference between the ANTH and CTRL scenarios for nine years between 1997-2001 and 2013-2017 and found that land-based inputs persistently compress aerobic and calcifier habitat capacity in the SCB during late summer, when capacity is at its seasonal minimum. A region 30-90 km from the mainland, southeast of Santa Catalina Island, experiences recurrent vertical compression of an average of 25%, but can be as much as 60%. DIN reductions predicted a reversal of habitat compression for both classes of taxa, with roughly equivalent habitat gains at both levels of reduction.

Understandably, more confidence exists in seawater chemistry predictions than in predicted changes in habitat capacity. The ocean acidification thresholds, derived though expert consensus of experimental and field data^50^, are in early stages of application and scientific and management acceptance^32, 48^. Meanwhile, the Metabolic Index application has a longer history of application in the peer-reviewed literature, has been validated with spatially-explicit abundance data, and is linked to population persistence^46, 51, 52^. The great similarity in predicted effects between O$_2$ and pH^25^ and in the reversal of those effects (this study) serve as multiple lines of evidence of ecosystem effects. While the modeled habitat compression associated with sublethal effects are recurrent features, considerable uncertainty exists in how this translates to population-level effects, particularly because the potential synergistic effects of acidification, suboptimal O$_2$, and warming as multiple stressors^53, 54^ and the mitigating effect of changing food availability^55^ are not included in our metrics.

In open embayments such as the SCB, a key control on eutrophication is the degree to which the mesoscale^56–58^ and submesoscale cross-shelf, high-frequency transport mechanisms^59, 60^ and accumulation of nitrogen and new organic matter in persistent eddies^61, 62^ exacerbate O$_2$ and pH loss^24^ and associated habitat compression^25^. Our offshore transport budgets suggest that progressive water recycling increases the horizontal flux of DIN primarily through eddy transport^24^. With progressive reductions in DIN loading in combination with water recycling, the risk that increased organic matter accumulation and subsurface O$_2$ and pH loss will occur has been dampened, regardless of these non-linear interactions of outfall plumes with these submesoscale, high-frequency transport mechanisms.

While transport budgets in each of the treatment scenarios can clarify the modification of NPP and respiration rates, more work is needed to better understand the non-linear interactions between meso- and submesoscale circulation features with ocean discharge of freshwater plumes^24, 63^. Analysis of nitrogen transport fluxes shows non-linear effects on the export of ammonium from the shelf to the offshore region. The bulk export is altered because of the net decrease of loading as water recycling increases (Table 1); however, the vertical shape of the fluxes suggests a different pathway of export (Fig. 7). In the case of 85% DIN reduction, 90% recycling, one would expect the ammonium transport to be concentrated and compensated at depth, yet there is significant total decrease in export (Fig. 7c). Some combination of nutrient management and water recycling may represent a “tipping point”, leading to decreased overall export. Further scenarios are needed to better understand the relationship and potential optimization between plume properties and export fluxes.

**Table 1:** List of model scenarios, conducted from August 2015 to October 2017, and flow-weighted average concentration and loading of each scenario across all treatment plants.

Summary of DIN Inputs per Scenario						
Scenario	Inorganic N	% Outfall	NH$_{4}^{+}$	NO$_{3}^{-}$+NO$_{2}^{-}$	DIN	Load
	Management	Volume Recycled	(mg/L)	(mg/L)	(mg/L)	(kg DIN/day)
CTRL	N/A	N/A	N/A	N/A	N/A	N/A
ANTH	2015-2017 loads	2015-2017 volumes	37.9	3.4	41.3	5706
Scenario 1	50% DIN reduction	0% recycling	7.6	14.1	21.7	3004
Scenario 2	50% DIN reduction	50% recycling	11.5	20.4	31.9	2804
Scenario 3	50% DIN reduction	90% recycling	23.2	39.4	62.6	2674
Scenario 4	85% DIN reduction	0% recycling	1.1	4.4	5.5	767
Scenario 5	85% DIN reduction	50% recycling	1.7	6.4	8.1	711
Scenario 6	85% DIN reduction	90% recycling	3.4	12.4	15.8	674

Several mechanisms are at play that may explain the non-linear patterns predicted in this study. Outfall plumes are subject to mixing and advection from the ambient ocean. Submesoscale and mesoscale structures are ubiquitous in the SCB^62, 64^ and affect far field fate and transport of effluent^17, 65^. Eddy flows are the primary dictator in effluent transport^17, 24^. Near the outfall, submesoscale stirring and straining dilutes the effluent plume and makes the local concentration highly variable. Further from the source, mesoscale meanders stir and advect the concentration field^17^. These dynamics acting on the effluent plume, in combination with changing density and volume of outfall discharge with potable water recycling, can cause the fate and transport, and thus the ecological response, to be non-linear.

In the Southern California Bight, interannual variability of ocean state is clearly a strong control that modulates the response of the system to DIN reductions^10^. During high productivity years (1999, 2017), the efficacy of anthropogenic nitrogen reductions on changing NPP is dampened relative to low productivity years by approximately an order of magnitude, driven by anomalous oceanic-atmospheric interactions that lead to a shallower nitracline and a nutrient-enriched surface mixed layer^66^. Thus, while coastal ecosystem eutrophication responses to point sources are more reliable than to non-point sources^40^, others have noted that complexities exist in the responses of coastal ecosystems to eutrophication^33, 67^, leading to some uncertainty in the success of management. Meanwhile, how climate change may affect the likelihood and strength of high productivity years in the future in the California Current System is uncertain. Models in the CCS have little agreement in the direction and magnitude that NPP will be altered with climate change^68, 69^. Moreover, intrinsic variability in numerical ocean modelling may play a role in variable spatial responses of each simulation (e.g., differing area of decreased NPP for Fig. 2d-e for the same freshwater input). Small perturbations and mesoscale turbulence can cause eddies to shift their position and lead to different spatial distribution of tracers^70, 71^. Despite eddy-induced differences in spatial distribution, the averaged results are consistent with the conclusions. Work to quantify this intrinsic variability of the model is underway.

This study only modeled two years for water recycling scenarios; this duration of simulations may not be enough to understand long-term effects of input changes. Longer simulations targeting nitrogen management alone show that climate variability modulates the effects of nutrient inputs (Fig. 2; Supplementary Fig. S1). Mitarai et al. (2009)^72^ corroborates that two years is sufficient. They quantified connectivity and residence time in the SCB using ROMS by simulating releases of Lagrangian particles at the SCB coast. They found that a majority (>80%) of Lagrangian particles from the SCB coast leave the domain within 90 days, which also supports our choice of three months of spin-up time. Regardless, it will be important to understand whether our distinct findings for 85% DIN reduction, 90% water recycling scenario hold under longer simulations. Furthermore, the horizontal and vertical distribution of the wastewater plumes at the outfall sites, which are parameterized in the 300-m simulations^30^, were not altered under progressive water recycling. This is intentional because that would have introduced a confounding factor; however, additional work needs to target how plume dispersion for individual outfalls will change under realistic conditions, and how this could impact the fate and transport of nitrogen^18^. Another variable to consider in making scenarios more realistic is that POTW agencies may undergo outfall management and modifications in response to water recycling that alter initial dilution, plausibly counteracting the effects of higher DIN concentrations due to water recycling. Idealized scenarios for individual POTWs may differ because of factors such as mechanical differences between outfalls not accounted for here.

These findings represent the first-of-their-kind to investigate the regional effects of outfall management on coastal eutrophication, but the scenarios represent a one-size-fits-all approach, with no attempt to optimize the synergy between DIN management and water recycling, nor attempt to quantify the full life cycle environmental impacts of advanced nutrient removal^16^. Furthermore, site-specific realities will dictate the feasible goals for water recycling as well as what could be achieved through nitrogen management. Additional work is needed to consider how to optimize nitrogen management vis-à-vis water recycling at sub-regional scales, as the location of discharge (e.g., near headlands that cause wakes) can strongly control mixing and dispersion by currents, eddies, fronts, and filaments. Palos Verdes and Point Dume in particular experience more intense circulation due to their bathymetry^73, 74^ when the northward current encounters topographic peaks. For this reason, some outfalls may have a disproportionate role in coastal acidification, hypoxia and offshore habitat loss, and this aspect has not yet been explored. Other outcomes, such as the risk of toxic harmful algae blooms (HABs), should be considered as a third lines of evidence, as water recycling will modify not only the dispersion, but also the magnitude of DIN concentrations and phytoplankton biomass in the nearfield (Table 1), a factor linked to the risk of HABs^75^. Furthermore, other bioremediation solutions such as kelp and bivalve aquaculture may provide a partial solution that could be a more cost-effective means to treat localized discharges from outfalls and nearby non-point source discharges^76, 77^. Numerical modeling feasibility studies could provide a pathway for quantitative nutrient trading and carbon crediting^78^.

Finally, work is needed to downscale and properly resolve fine-scale pH and O$_2$ variability under future climate scenarios^46^, ranging from no action to strong mitigation, in order to project the range of possible pH, O$_2$, and warming stressors that coastal ecosystems will experience. This would provide an understanding of how much benefit these management actions may confer to increased coastal resilience given accelerating acidification and deoxygenation from climate change^79^ and provide key context to further inform the role of coastal water quality management in climate change adaptation.

## Methods

### Overview of approach

We utilize the Regional Ocean Modeling System (ROMS)^80, 81^, coupled to the Biogeochemical Elemental Cycling model (BEC)^28, 82^, and configured for the California Current System (CCS) to simulate the effect of changing ocean outfall characteristics from a set of idealized scenarios of water recycling and nitrogen management, applied uniformly across the 23 wastewater treatment plants between August 2015 and October 2017. We evaluated spatial and temporal changes in NPP, respiration rate, oxygen, and pH^24^. We then utilized habitat capacity metrics for key marine pelagic taxa to evaluate whether changes in acidification and oxygen^25^ translate to potential biological effects.

### Study area

The SCB is a marine open embayment, approximately 94,000 km$^2$ in size, that spans from Point Conception (34.45$^{\circ }$N) to Baja California, Mexico (32.53$^{\circ }$N). The climate of this region is Mediterranean, with rainfall concentrated largely over the winter months of December-March. The majority of runoff to the SCB occurs during wet weather storm events, with an average of 10-15 rain events per year^83^. Precipitation in this region has strong inter-annual variability; over the period of 1997-2017, total median annual rainfall was 9.9 cm, with ranges of 6.3-11.8 cm representing the 25th and 75th percentile and an annual maximum of 157 cm.

The SCB receives point source, non-point sources, and natural discharges from 75 rivers and 19 ocean outfalls, in addition to atmospheric deposition. Anthropogenic nutrient sources, which represent 98% of coastal nitrogen exports^13^, rival natural upwelling in magnitude^84^, roughly doubling available nitrogen to nearshore coastal waters. The dominant contribution is from point sources discharged to ocean outfalls, representing 92% of total nitrogen discharged from land-based sources^13^. Among facilities permitted by the U.S. National Pollution Elimination Discharge System (NPDES), POTWs comprised the majority of discharges that occur via outfalls to the SCB. The four largest facilities each discharge over 100 million gallons per day, and account for 86% of the total POTW effluent volume. These facilities are the Hyperion Treatment Plant (HTP) operated by the City of Los Angeles in the Santa Monica region, the Joint Water Pollution Control Plant (JWPCP) operated by the Los Angeles County Sanitation Districts in the San Pedro region, Orange County Sanitation District (OC San) Reclamation and Treatment Plants in Orange County, and the City of San Diego’s Point Loma Wastewater Treatment Plant (PLWTP) in South San Diego (Fig. 1). HTP, JWPCP, and OC San currently treat to secondary or advanced secondary levels, while PLWTP treats to primary level. The small POTWs constitute a combination of secondary or tertiary treated effluents. One large and several small POTWs have partial or full nitrification-denitrification^85^, which decreases DIN concentrations and loads. The historical record captures the changes in effluent volume and constituent concentrations with progressive wastewater treatment upgrades^13^. The large POTW ocean outfalls discharge 2.5 to 8 km offshore at depths of 60 to 90 m below the sea surface, while the small POTW outfalls discharge approximately 1 to 2.5 km offshore between 5 and 40 m water depth (Fig. 1).

### Numerical ocean model

ROMS-BEC^80–82^ was adapted and validated for the CCS^28, 29^. Ocean hydrodynamics are modeled with ROMS, a free-surface, terrain-following coordinate model with 3-D curvilinear coordinates that solves the primitive equations with split-explicit time steps. It contains state-of-art numerical algorithms that provide an accurate and stable representation of physical processes down to scales of hundreds of meters or less, and allows for offline downscaling of high-resolution subdomains within larger domains^28, 30^.

ROMS is dynamically coupled to the BEC model^28, 82^. BEC is a multielement (C, N, P, O, Fe, and Si) and multiplankton model that includes three explicit phytoplankton functional groups (picoplankton, silicifying diatoms, and N-fixing diazotrophs), one zooplankton group, and dissolved and sinking organic detritus^28^.

The CCS configuration of the ROMS-BEC model domain scales from a 4 km horizontal resolution configuration, CCS-wide, to a 1 km resolution grid covering much of the California coast (latitude <40.25$^{\circ }$N), to a 0.3 km grid in the SCB (Fig. 1), where investigations of local anthropogenic inputs have been focused. For investigations of coastal eutrophication within the SCB, simulations at 0.3 km horizontal resolution are used in order to adequately capture the effect of submesoscale circulation and finer horizontal bathymetric features on physics and nutrient transport^30, 59, 60^. The SCB 0.3 km model simulations are forced with a monthly time series of spatially-explicit terrestrial inputs, including freshwater flow, nitrogen, phosphorus, silica, iron, and organic carbon representing natural and anthropogenic sources^13^. These data include monitored POTW ocean outfalls and riverine discharges (2016-2017). POTW effluent data were compiled from permit monitoring databases or directly from POTWs. Monthly time series of surface water runoff from 75 rivers are derived from model simulations and monitoring data^13^.

Simulations conducted with the 4 km ROMS-BEC model domain have been validated for regional-scale atmospheric forcing, physics, and biogeochemistry, including O$_2$, carbonate saturation state, primary productivity, and hydrographic parameters, demonstrating that the model captures broad patterns of physical and biogeochemical properties in the CCS^28, 29^. Additional focused validation of nearshore, anthropogenically-enhanced gradients in nutrients, primary production, oxygen, and pH in model simulations has been conducted at 0.3 km resolution to document model utility to investigate the impacts of coastal eutrophication on acidification and oxygen loss^30^.

### Modeling scenarios

Modeling scenarios included “experimental treatment” of reduction in outfall DIN concentration and percent of outfall volume recycled for potable reuse (Table 1). These scenarios represent a bracket of effects that range from current day land-based loading (ANTH) to a combination of nitrogen management and outfall discharge recycling scenarios (Scenario 1-6) to “no land-based loading”, representing ocean only (CTRL)^10^. All scenarios included a background suite of inputs, spatially-explicit inputs of rivers, outfalls, and global CO$_2$, but experimental treatments were only applied to the 23 treatment plants. Each model simulation was run for a total of 27 months during the period of August 2015-October 2017 with the first three months as spin-up time. These two years represent bookends in coastal ocean productivity, thus allowing us to understand the range in response across ocean state. To further understand how interannual variability in climate phase and ocean state affects coastal ecosystem response to nitrogen loading, the ANTH and scenarios 1 and 4 were repeated during August 1997-October 1999 period with August 2015-October 2017 terrestrial inputs, a period that overlaps with documented model performance^30^ and includes an El Ni$\mathrm {\tilde{n}}$o/La Ni$\mathrm {\tilde{n}}$a cycle. Output was stored as daily averages.

Scenarios 2, 3, 5, and 6 represent scenarios in which a portion ranging from 50% to 90% of the volume of the effluent currently being discharged to the outfall is recycled for potable reuse. In these scenarios, the final effluent outfall volume and constituent concentration was calculated for each individual outfall by a set of assumptions that governed the efficiency of water treatment and water recovery that were applied uniformly across all outfalls. These assumptions were informed by literature sources and current operating parameters at an existing potable water recycling program in the region^27^:

1. Water recovery efficiency of potable water recycling is 80%, meaning 20% of any given outfall volume that is diverted from the outfall for water treatment would return to the outfall after treatment^86, 87^. A target of 50% recycled means that 40% of the total outfall volume is reduced, with 10% of the volume not recycled and returned to the outfall.
2. Concentrate added to return volume is derived from reverse osmosis permeate concentration, which is a function of membrane recovery efficiency. Recovery efficiencies were established based on polyamide recovery efficiencies in published literature^88–91^. Specific recovery efficiency varied by effluent constituent as follows: 85% removal of NO$_{3}^{-}$ and NO$_{2}^{-}$, 95% removal of ammonium, phosphate, alkalinity, and silicate, 97% removal of organic carbon, organic nitrogen, and organic P, and 100% removal of salt and dissolved iron. High removal efficiencies of DIN forms resulted in the nitrogen loading staying roughly the same across the tier of 50% (scenarios 1-3) or 85% DIN reduction scenarios (scenarios 4-6), while volumes declined and DIN concentrations in the final effluent increased.

The changes to the terrestrial inputs for ocean outfall concentration and volume were calculated based on the NPDES permitted discharges for each of the 23 POTWs. Briefly, effluent DIN concentrations were held constant (ANTH; $\sim$37.9 mg/L NH$_{4}^{+}$ and 3.4 mg/L NO$_{3}^{-}$+NO$_{2}^{-}$), or uniformly reduced to represent DIN reductions of 50% (7 mg/L NH$_{4}^{+}$ and 13 mg/L NO$_{3}^{-}$+NO$_{2}^{-}$; scenarios 1-3) or 85% (1 mg/L NH$_{4}^{+}$ and 4 mg/L NO$_{3}^{-}$+NO$_{2}^{-}$; scenarios 4-6), representing DIN management from partial to full nitrification-denitrification (Table 1). Outfalls that already met targeted DIN concentrations were held constant.

Supplementary Table S2 shows average flows, loads, and flow-weighted mean DIN concentrations as a function of increased water recycling at each given level of nitrogen management across all 23 treatment plants and summarized in Table 1. Note that these are idealized scenarios where effluent characteristics are altered consistently across all agencies; thus, Supplementary Table S2 does not reflect how individual POTW agencies may change their effluent characteristics or outfall management in the future. Table 1 shows that loading and concentrations of the scenarios do not exactly reflect the DIN reduction percentages. The reasons are because these calculations are flow-weighted values; some treatment plants have different percentages of DIN reduction because they are close to or have met the target inorganic nitrogen management already; and recycling removes a small portion of inorganic nitrogen due to membrane recovery efficiency. Potable water recycling alone does not necessarily imply any significant nutrient reduction, and individual POTW decisions to manage nutrients are secondary.

### Scenario analysis methods

We rely on land-based change assessment methodologies that have previously been established^10^ and further expanded on to include a mechanistic biogeochemical mass balance analysis^24^ and an assessment of the effects of subsurface acidification and oxygen loss on habitat capacity for marine calcifying and aerobic taxa^25^.

#### Mass balance analyses

Biogeochemical mass balance analyses were used to answer questions (1) and (2) with the goals of describing changes in the NPP, respiration, O$_2$, and pH. NPP is calculated online in BEC^28^. The mass balance analysis for O$_2$ includes physical transport and biogeochemical processes and is used to calculate respiration^24^.

Model output of dissolved inorganic carbon, alkalinity, silicate, phosphate, temperature, and salinity are used to calculate pH using CO2SYS^92^. Changes in NPP and respiration rate link directly to subsurface O$_2$ and pH decrease and for this reason serve as focal variables for the change assessment. Preliminary analyses illustrated land-based inputs altered rates of respiration between 0-600m, but most changes occur between 50-150 m. For this reason, monthly averages of the biogeochemical states, rates, and transport fluxes were calculated for water column depths between 0 and 200 m.

#### Horizontal export of ammonium from the shelf

Kessouri et al. (submitted)^24^ identified that the mean and eddy horizontal flux of ammonium across the 200 m isobath was a key determinant of NPP increase and O$_2$ and pH reduction in offshore basins. We utilize ammonium transport as indicator to investigate how decreasing water volumes may impact the transport and fate of anthropogenic nutrients offshore. This transport is quantified across an alongshore vertical section that goes from the Mexican border to Goleta in Santa Barbara County and from 0 to the bottom (200 m depth)^31^. The calculation is conducted using monthly averaged fields of velocity and concentration of ammonium. Transport from the shelf to the offshore region is shown by positive values; transport from the offshore region to the shelf is shown by negative values.

#### Metrics to evaluate changes to aerobic and calcifier habitat capacity

To evaluate the potential biological effects of subsurface acidification and oxygen loss, we used the methodology of Frieder et al. (submitted)^25^ to quantify changes in habitat capacity for aerobic taxa and for marine calcifiers. The concept of change to habitat capacity is based on the premise that marine organisms are directly influenced by environmental gradients in temperature, oxygen, and pH–factors that have been clearly implicated in shifting species distributions^46, 55, 93, 94^. In the Southern California Bight, Frieder et al. (submitted)^25^ found changes to the vertical thickness of aerobic and calcifier habitat capacity attributable to eutrophication. A region of annually recurring habitat compression is most pronounced 30-90 km from the mainland, southeast of Santa Catalina Island. The goal of this analysis is to document how this predicted habitat compression is altered in the nitrogen management and water recycling experiments.

For the effects of subsurface acidification on calcifier habitat capacity, we calculate the vertical thickness of optimal aragonite saturation state ($\Omega _{\textrm{Ar}}$) conditions. A value of $\Omega _{\textrm{Ar}}$ of 1.4 is used to define the condition below which sublethal organismal responses have been documented to commonly occur, in particular for calcifying zooplankton (e.g., pteropods)^50, 95, 96^. Pteropods are ubiquitous, holoplanktonic calcifiers that have a well-documented, specific sensitivity to ocean acidification and serve as an important prey group for the diet of ecologically and economically important fishes, birds, and whales^97–99^. Notably, Frieder et al. (submitted)^25^ found that the persistence of habitat compression is relatively insensitive to the choice of threshold down to a value of approximately 1.1 in the ANTH simulations.

For the effects of subsurface oxygen loss on aerobic habitat capacity, we rely on the mechanistic framework of the Metabolic Index ($\Phi$)^51, 52^. This approach integrates the sensitivity of metabolism to the combined effects of O$_2$ and temperature. $\Phi$ is defined as the ratio of O$_2$ supply to resting demand. We calculate the habitat thickness for which $\Phi$/$\Phi _{\textrm{CRIT}}$ $\ge$ 1, a value below which demarcates environment in which an organism can sustain enough active energetic demands to maintain viable populations. Following the methodology of Frieder et al. (submitted)^25^, we use metabolic traits for the northern anchovy (*Engraulis mordax*) as an indicator species because (1) habitat range predicted by the Metabolic Index has been validated with abundance data documenting their biogeographic distribution in the southern CCS^46^; (2) northern anchovy represents the 75th percentile of known oxygen sensitivity traits for the CCS^46^ and (3) adult anchovy have historically been observed within 30 km offshore and from 0 to 100 m water depth^100^, a habitat range that aligns with the location of documented effects on seawater chemistry from land-based nutrient inputs^24^.

### Supplementary Information

Supplementary Information 1.

Supplementary Information 2.

## Data Availability

The datasets used and/or analysed during the current study are available from the corresponding author upon reasonable request.
