# Supplementary material for: Effect of ocean outfall discharge volume and dissolved inorganic nitrogen load on urban eutrophication outcomes in the Southern California Bight
Source: Sci Rep. 2023 Dec 13;13:22148. doi: 10.1038/s41598-023-48588-2 (PMC10719394; doi:10.1038/s41598-023-48588-2)
Supplement: Supplementary file 1 — Supplementary Information 1. [file 41598_2023_48588_MOESM1_ESM.pdf]

Supplementary Tables

		O_2 mmol m^{-3}	pH
	Mean	0.828	0.0016
50% N Red.	5%	-1.7	-0.003
	95%	5.011	0.01
	Mean	0.107	0.0002
50% N Red. 50% Recy.	5%	-5.361	-0.0102
	95%	3.709	0.0069
	Mean	0.219	0.0005
50% N Red. 90% Recy.	5%	-2.938	-0.0057
	95%	3.729	0.0072
	Mean	1.083	0.0021
85% N Red.	5%	-2.177	-0.0037
	95%	4.851	0.0091
	Mean	0.097	0.0004
85% N Red. 50% Recy.	5%	-4.806	-0.0091
	95%	3.025	0.0056
	Mean	1.071	0.0019
85% N Red. 90% Recy.	5%	-1.897	-0.0038
	95%	4.205	0.0076

Table S1. Average change in absolute, 5th, and 95th percentile values in O_2 and pH Bightwide in mmol m^{-3} for each scenario – ANTH between November 2015 and October 2017.

Please download the Excel .xlsx file online.

Table S2. Changes in average flows, loads, and flow weighted mean DIN as a function of increased water recycling at each given level of nitrogen management across all 23 treatment plants.

Supplementary Figures

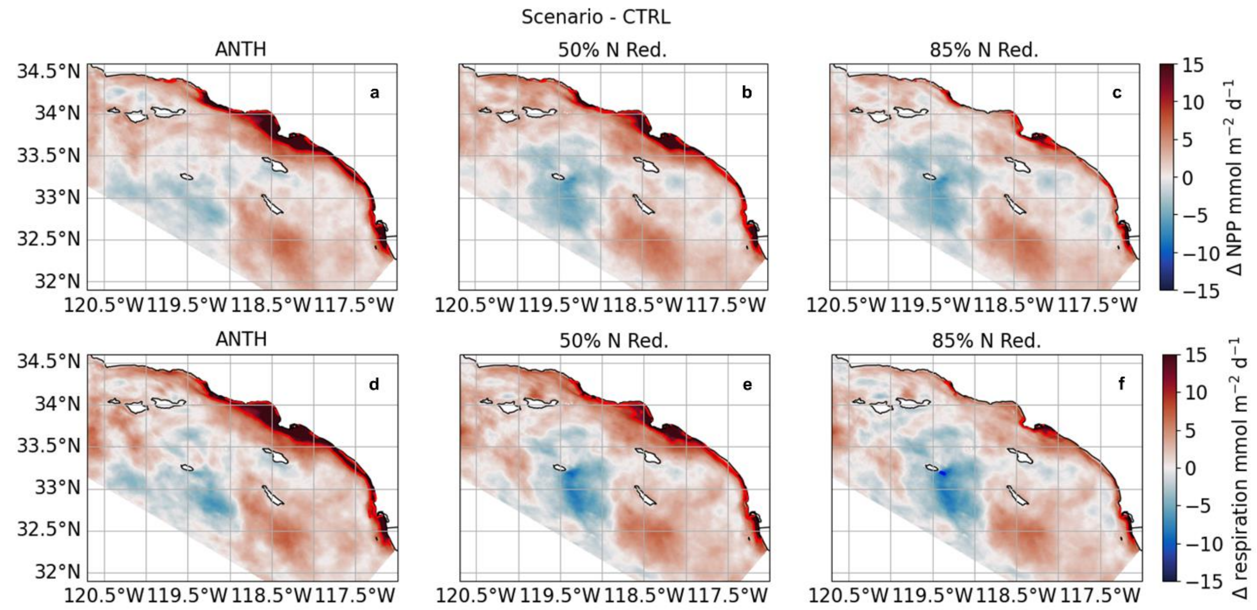

Figure S1. As Figure 2c-h maps for November 1997 – October 1999.

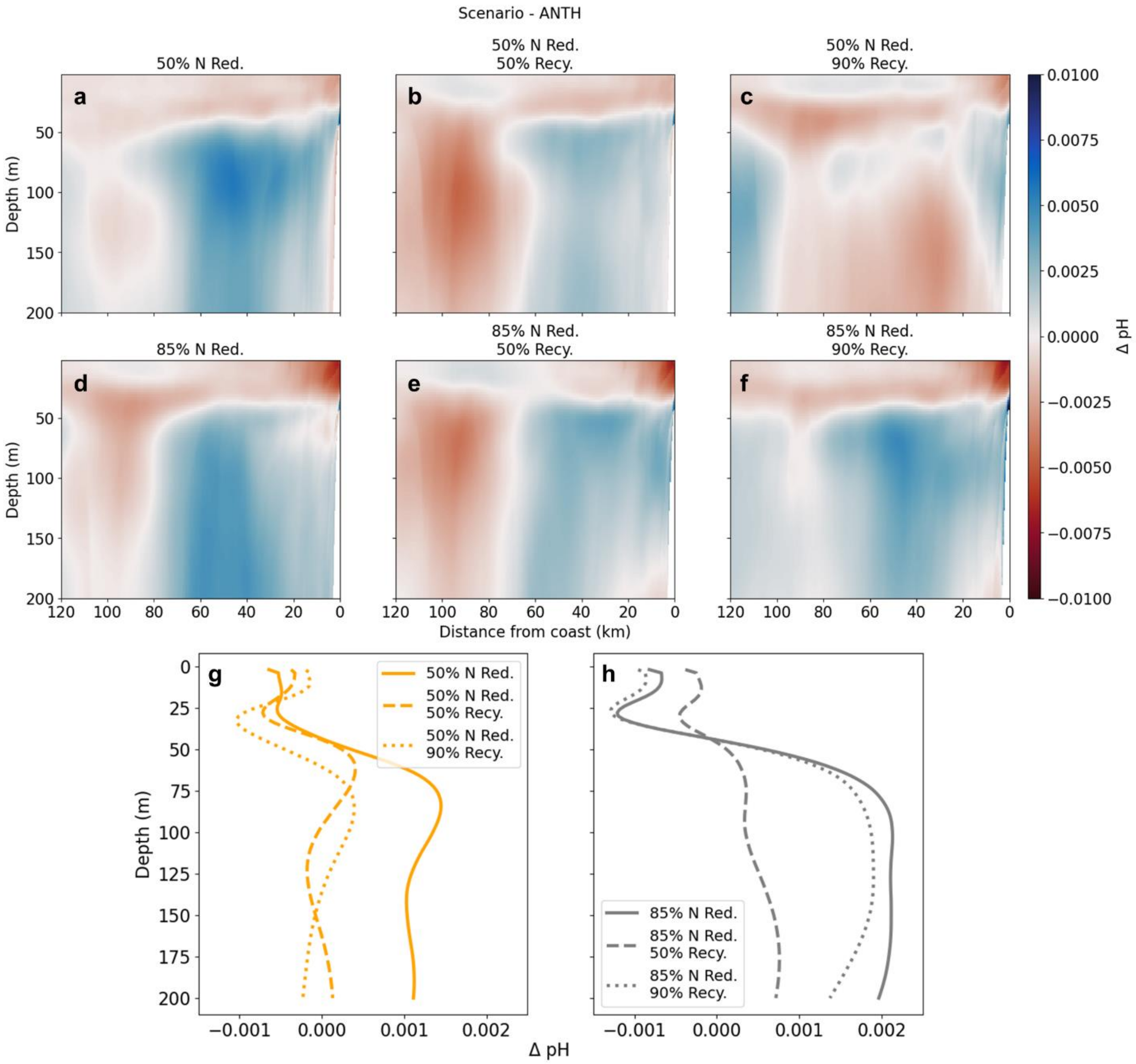

Figure S2. As Figure 3 for pH.

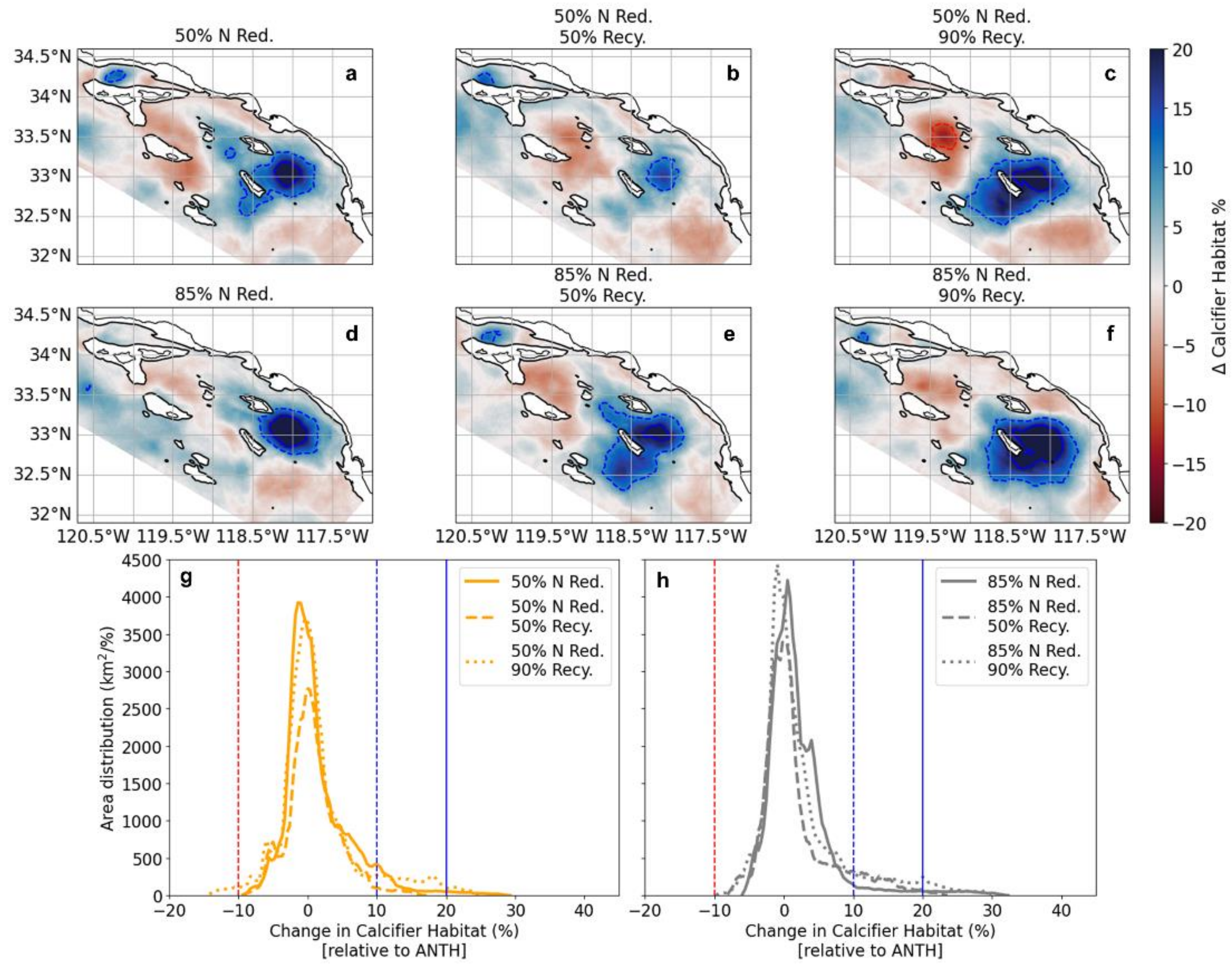

Figure S3. As Figure 4 for calcifier habitat.
